# Outcome after mitral valve surgery for functional mitral regurgitation in idiopathic dilated cardiomyopathy

**DOI:** 10.1186/1749-8090-8-S1-O292

**Published:** 2013-09-11

**Authors:** V Shumavets, A Ostrovski, I Andraloits, S Kurganovich, I Grinchuk, D Romanovski, O Jdanovich, Y Ostrovski

**Affiliations:** 1 Cardiac Surgery Department, Belarus Cardiology Centre, Minsk, Belarus

## Background

To assess the outcome of surgery of functional mitral regurgitation (FMR) in idiopathic dilated cardiomyopathy (IDCM).

## Methods

We retrospectively investigated 146 patients (131 men, 16 women; mean age, 67 years) with severe functional mitral valve (MV) insufficiency due to dilated cardiomyopathy that were operated on between 2004 till 2012. 35 pts were in New York Heart Association class IV and 102 were in class III. Most of pts had severe enlarged LV (mean end-diastolic size 73.7±7,1ml and volume 293±80ml) with depressed systolic function (mean ejection fraction – 26.1±5,6%). Concomitant procedures were tricuspid repair (139 pts) and atrial fibrillation ablation (32 pts). In 35 pts (24%) epicardial LV lead was fixed with subsequent CRT implantation during the same hospitalization. Mitral valve annuloplasty with different types of undersized ring were performed in 101 patients, and 45 had mitral valve replacement (in 6 pts mechanical and in 39 pts biological).

## Results

There were no perioperative deaths, in 2 pts BiVAD were started due to inability to wean from CPB. 4 patients (2.7%) died within 30 days postoperatively. Overall freedom from MR ≥ 2+ was 96.1%. Both LV end-diastolic and end-systolic volumes indexed significantly decreased (both p=0.0001). After MV repair ejection fraction increased from 25.2±7,5% to 29,2±9,4% (p=0,04). The mean follow-up was 47.6±4.1 months. 1-years survival was 78±0.04%, 5-years - 39±0.08%. The fact of mitral valve replacement versus repair did not influence survival (long-rank p=0,145). The type of ring used for annuloplasty not influenced survival (long-rank p=0.37). Statistical analysis identified preoperative NYHA class as predictor of long-term survival (hazard ratio (HR) 4.53, 95% CI 2.7–13.5, p=0.004).

## Conclusion

Despite high operative risk in patients with IDCM, mitral valve surgery can be performed successfully with acceptably low hospital mortality, clinical improvement and a moderate recovery of LV function.

